# LncRNA, an Emerging Approach for Neurological Diseases Treatment by Regulating Microglia Polarization

**DOI:** 10.3389/fnins.2022.903472

**Published:** 2022-07-04

**Authors:** Xiaoyu Gao, Zilong Cao, Haifeng Tan, Peiling Li, Wenen Su, Teng Wan, Weiming Guo

**Affiliations:** ^1^Sports Medicine Department, Huazhong University of Science and Technology Union Shenzhen Hospital, The 6th Affiliated Hospital of Shenzhen University Health Science Center, Shenzhen, China; ^2^Hengyang Medical College, University of South China, Hengyang, Hunan, China

**Keywords:** microglia polarization, lncRNA, miRNA, neurological diseases, neuroinflammation

## Abstract

Neurological disorders cause untold human disability and death each year. For most neurological disorders, the efficacy of their primary treatment strategies remains suboptimal. Microglia are associated with the development and progression of multiple neurological disorders. Targeting the regulation of microglia polarization has emerged as an important therapeutic strategy for neurological disorders. Their pro-inflammatory (M1)/anti-inflammatory (M2) phenotype microglia are closely associated with neuronal apoptosis, synaptic plasticity, blood-brain barrier integrity, resistance to iron death, and astrocyte regulation. LncRNA, a recently extensively studied non-coding transcript of over 200 nucleotides, has shown great value to intervene in microglia polarization. It can often participate in gene regulation of microglia by directly regulating transcription or sponging downstream miRNAs, for example. Through proper regulation, microglia can exert neuroprotective effects, reduce neurological damage and improve the prognosis of many neurological diseases. This paper reviews the progress of research linking lncRNAs to microglia polarization and neurological diseases.

## Introduction

Microglia, as the major intrinsic immune cells in the brain, play an important role in maintaining the homeostasis of the brain’s internal environment (Matcovitch-Natan et al., 2016). Under physiological conditions, the number of microglia in the brain of adult mice and humans remains stable throughout life (Askew et al., 2017). But in some cases, their number is significantly altered in certain neurological diseases, such as Parkinson’s disease (PD). Under pathological conditions, microglia will undergo a series of morphological and functional changes, including proliferation, morphological changes, migration and phagocytosis. There are many studies based on microglia to study disease onset, progression, and prognosis. An in-depth study of microglia polarization revealed that M1-phenotype microglia promote neuroinflammation and thus exert neurotoxic effects (Kwon and Koh, 2020). After stimulation by a variety of microorganisms, microglia polarize to the M1 phenotype, inducing neuroinflammation and cellular damage, and also have been found to be associated with the development of several neurodegenerative diseases (Mathy et al., 2021). It has been found that induction of M2 polarization can alleviate neurological disorders such as ischemic stroke, axonal injury, and PD (Wang et al., 2018). These have brought microglia into the limelight.

The non-coding RNAs (ncRNAs) has been extensively studied recently because they are transcribed but not translated. These non-coding transcripts are not junk noise; they are biologically important. NcRNAs include small ncRNAs [mainly microRNAs (miRNAs)] and other non-coding transcripts of less than 200 nucleotides. LncRNAs are novel non-protein-coding transcripts of more than 200 nucleotides (Kapranov et al., 2007). LncRNAs play an important role in regulating various cellular activities by improving brain development, neuroplasticity and promoting neuroprotection. LncRNAs are involved in a variety of cellular physiological processes such as apoptosis, cell cycle regulation, cell differentiation and information transduction (Ni et al., 2020). Depending on the location of the encoded genes, the transcriptionally generated lncRNAs have different functions. In general, lncRNAs can regulate the expression and function of miRNAs. MiRNAs can in turn bind to lncRNAs and can regulate their stability (Zhao Z. et al., 2020). The more studied regulation of lncRNAs is derived from the ceRNA hypothesis, which states that lncRNAs can weaken the effect of miRNAs by competitively binding to the downstream substrate mRNA of miRNAs. At the same time, lncRNAs can also affect the transcriptional level of genes through DNA methylation (Lu et al., 2020).

LncRNAs are tissue-specific regulators that direct cell differentiation and functional transformation. LncRNAs were found to regulate microglial cell activity. It has been found that lncRNAs can affect microglia and thus the progression of central nervous-related diseases through a variety of mechanisms. Studies in patients with epilepsy have found that lncRNAs can affect microglia activation by influencing certain signaling pathways or miRNAs (Han et al., 2018a; Xiaoying et al., 2020). When studying multiple sclerosis (MS) using a mouse demyelination model, lncRNAGAS6 can affect the progression of experimental autoimmune encephalomyelitis by regulating microglia polarization and promoting remyelination (Sun et al., 2017). NLR-family CARD-containing protein 4 (NLRC4) protein is associated with microglia apoptosis. In a mouse model of diabetic brain ischemia-reperfusion, lncRNA Fendrr promotes microglia apoptosis by inhibiting NLRC4 degradation and thus reduces the series of inflammatory injuries triggered by microglia activated in a hypoxic environment (Lv et al., 2016; Wang et al., 2021). To elucidate the link between lncRNA, microglia and central neurological diseases, this paper reviews the effects of lncRNAs on microglia and highlights the effects of lncRNAs on a variety of central neurological disorders by regulating microglia polarization.

## Overview of Microglia

### Origins, Biological, and Pathological Characteristics of Microglia

Currently, we believe that microglia are mainly derived from embryonic yolk sac progenitors and to a lesser extent from peripheral microglia-like cells replenished by circulation (Ginhoux et al., 2010; Ginhoux and Garel, 2018). There are two accounts of the replenishment of microglia populations. Some studies point out that the regenerated microglia actually come only from the regeneration of residual microglia and not from peripheral blood, astrocytes or other neurons (Huang et al., 2018). However, 2% of microglia may also be replaced by foreign cells through conventional bone marrow transplantation. Thus, microglia replacement therapy may provide clues for relevant central system treatments. However, conventional gene therapy may show the limitation that the range of action of viral vectors is too small. Therefore, how to make exogenous microglia replace the original microglia becomes the bottleneck of gene therapy. However, the results of conventional transplantation are not satisfactory (Derecki et al., 2012). Therefore, Bo-Peng et al. now propose methods to improve the efficiency of microglia replacement, making it possible to replace microglia throughout the nervous system or in specific parts of the brain region as a possible treatment (Xu et al., 2020c). Microglia enter the central nervous system (CNS) during development and have an important impact on the maturation and shaping of the entire nervous system. The main roles of microglia include sensing environmental changes, promoting normal neuronal function, and maintaining neural microenvironmental homeostasis. Microglia branches cover most of the brain and can transform into streptomyces, amoeba-like structures to engulf and remove cells or other structures from damaged areas of the brain, thus maintaining normal nervous system function (Tam and Ma, 2014). In addition, microglia affect synaptic density and connectivity by phagocytosis of neurons that do not establish functional circuits, which is closely related to cognitive function (Sierra et al., 2010; Paolicelli et al., 2011). Studies have reported that microglia can release superoxide ions that promote apoptosis of hippocampal neurons. This promotion of apoptosis of damaged or malfunctioning hippocampal neurons has a positive effect on the regeneration of neuronal cells in the hippocampal region (Marín-Teva et al., 2004; Diaz-Aparicio et al., 2020). Microglia can defend against infectious pathogens by expressing multiple receptors such as chemokine receptors and receptors for neurotransmitters and releasing cytokines such as inflammatory factors to initiate neuroinflammation.

Microglia are associated with the development of a variety of disorders, including social deficits, impaired language development, mental retardation, motor abnormalities, and may lead to autism, schizophrenia, obsessive-compulsive disorder, etc. (Zhan et al., 2014). Microglia activated by infectious agents produce pro-inflammatory factors that are associated with the development of depression (Yirmiya et al., 2015). In the infarct zone of ischemic stroke, microglia can perform biphasic functions of neural tissue damage or repair by secreting toxic substances and growth factors (Ma et al., 2017). In the early stages of Alzheimer’s disease (AD), microglia reduce neuronal damage from toxic-like amyloid by phagocytosis of Aβ (amyloid beta-protein). As the disease continues to progress, activated microglia mediate the phagocytosis of synapses by expressing C3a, C5a and C3R (C means Complement, R means receptor), leading to synaptic deficits (Hansen et al., 2018). The regulation of microglia activation has now become a therapeutic target for many diseases.

### Regulation of Microglia Polarization

Currently, microglia dichotomy is the conventional view of microglial cell polarization and there might be concept renewal in the white paper to understand the microglia states in the near future. Microglia are mainly divided into M1 pro-inflammatory (classically activated) and M2 anti-inflammatory (alternative activated) types. In addition, they can be further subdivided into M2a, M2b and M2c subtypes according to their different properties (Gensel and Zhang, 2015). M2A belongs to the typical healing and repair phase of microglia. It is usually induced by IL-4 or IL-13, which implies greater phagocytosis to remove cellular debris, along with the production of insulin-like growth factor-1 and the anti-inflammatory cytokine IL-10 (Pepe et al., 2014). M2b is transition activated and has immunomodulatory functions. It is activated by LPS or IL-1β, induced by immunoglobulin Fc receptor ligands, upregulates CD32 and TGFβ and downregulates IL-12 production. M2C is known as an acquired inactivated microglia and is usually induced by the anti-inflammatory cytokine IL-10 or glucocorticoids, which can shut down the immune response of microglia (Walker and Lue, 2015). Recent studies have also identified specific types of microglia such as MGnD and MG-dNF types that are closely associated with the pathogenesis of AD (Krasemann et al., 2017). However, some objections to the dichotomy have recently emerged. First, gene products of two and more polarization states have been detected in reactive microglia populations. This suggests that the phenotypic transformation of microglia may be context-dependent. The current dichotomy is influenced by the variability of single-cell gene sequencing and spatial transcriptomic results under various physiological and pathological conditions, preventing microglia from being simply classified as M1/M2 phenotype. However, a large number of studies are still based on microglia function and pro- and anti-inflammatory phenotypes, and are widely used in experimental studies. Pro-inflammatory genes such as H19, SNHG15, lncGAS5 are able to promote the M1 phenotype of microglia and anti-inflammatory genes such as SNHG3, lncRNAPeg13, lncRNA uc.80- are able to promote the M2 phenotype. Based on the paucity of studies against dichotomization, this simple function-based typing is still alive and has great potential to guide treatment (Ransohoff, 2016). M1/M2 of microglia are generally distinguished by their markers, which for M1 generally include CD16, CD32, CD86, iNOS, etc. Markers for M2 include CD206, Arg-1, etc. In general, microglia are in a dynamic equilibrium between the classical pro-inflammatory (M1 type) and anti-inflammatory (M2 type) types, and their transformation is related to the local microenvironment in which they are located. *In vitro*, microglia can be induced to convert to the M1 phenotype by LPS or IFN-γ, or to the M2 phenotype by IL-4 or IL-13 (Orihuela et al., 2016). Due to the transformable nature between different subtypes of microglia, it is possible to convert M1 phenotype microglia to M2 cells by various means. Many drugs have been shown to reduce damage *in vitro* by inducing their conversion to M2 cells. For example, Quercetin, which inhibits the polarization of microglia to M1 type, may be useful in treating spinal cord injury, while inducing their polarization to M2 type may be useful in diseases such as stroke (Fan et al., 2019).

The regulation of polarization in different types of microglia is dependent on different pathophysiological conditions and involves multiple signaling pathways. It was shown that in response to stimulation by pro-inflammatory factors, such as interleukin-1β (IL-1β) and IL-6, tissue proteinase C promotes microglia M1 polarization through activation of the ca2 + -dependent PKC/p38MAPK/nuclear factor κB (NF-κB) pathway (Liu et al., 2019). The Toll-like receptor 4 (TLR4) signaling pathway also mediates the polarization of M1 microglia. Bacterial lipopolysaccharide can bind TLR4 and its co-receptor CD14 to promote activation of caspase-8 and caspase-3, thereby promoting NF-κB activation and inducing an inflammatory response. Curcumin and candesartan reduce injury by inhibiting the TLR4/NF-κB pathway to promote microglia M2 polarization (Zhang et al., 2019d; Qie et al., 2020; Rodríguez-Gómez et al., 2020). The TLR4/NF-κB pathway is used to reduce damage to microglia. Oxidative stress promotes signal transducer and activator of transcription 1 (STAT1) phosphorylation and s-glutathionylation in BV2 microglia, which is associated with their M1 polarization (Butturini and Boriero, 2019). (Peroxisome proliferator-activated receptor-γ) PPAR-γ signaling also plays a role in polarization, and rosiglitazone, an activator of PPAR-γ, can promote microglia conversion to M2, while the PPAR-γ inhibitor GW9662 can inhibit its conversion (Wen et al., 2018). Activation of microglia TGFβ1-related signaling pathway inhibits the neurotoxic effects on dopaminergic neurons. TGFβ1 can be considered to play a role in inhibiting microglia M1 polarization (Spittau et al., 2020). TREM2 related microglia have recently been identified to be associated with neurodegenerative diseases. Inhibition of miR-155 expression during neurodegeneration by already apoptotic neurons and the Aβ amyloid plaque trigger receptor 2 (TREM2)-apolipoprotein E (APOE)-driven signaling pathway induces microglia to participate in neuronal damage (Keren-Shaul et al., 2017). TREM2 is an important negative regulator of neuroinflammation that both inhibits M1 activation and plays a key role in M2 polarization to reduce neural damage, and its mutation increases the risk of PD and AD (Krasemann et al., 2017; Zhang Y. et al., 2018). In summary, the polarization state of microglia is regulated by multiple signaling pathways that may be targets for the treatment of a variety of neurological diseases.

## LncRNA/Microrna Axis and Microglia Polarization

### MicroRNA and Microglia Polarization

**FIGURE 1:**
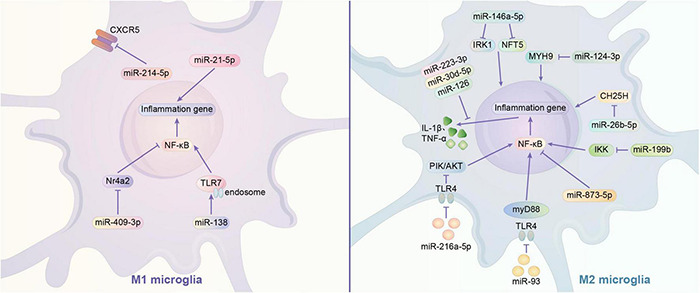
Regulation mechanism of miRNA on microglia polarization. (1) Signaling pathway of M1 polarization regulated by miRNA. miR-21-5p acts on microglia through releasing pro-inflammatory substances, while miR-214-5p suppress the expression of CXCR5 and other pro-inflammatory substances like IL-6, IL-8, TNF-α. NF-κB is thought to be a common pro-inflammatory signal pathway. miR-409-3p can downregulate Nr4a2 (a suppressor of NF-κB) to activate microglia. MiR-138 may activate the TLR7 to promote NF-κB. (2) Signaling pathway of M2 polarization regulated by miRNA. The exosomes containing miR-223-3p, miR-30d-5p, or miR-126 may suppress the synthesis of inflammatory factors to inhibit the activation of microglia. There is some genetic expression may influence the polarization of microglia.miR-146a-5p may downregulate IRK1 and NFT5 to inhibit the activation of microglia. MiR-124-3p may suppress the expression of MYH9.miR-26b-5p may suppress the expression of CH25H. miR-873-5p may suppress the NF-κB signaling pathway, which is very common in inflammation. All these three miRNA can inhibit microglia polarization. TLR4 signal pathway is a common way to promote inflammation, while the inhibition of it can suppress the process. Exosomes of miR-93 may suppress the NF-κB signaling pathway through downregulating TLR4 expression. Similar exosomes of miR-216a-5p may activates the PI3K/AKT signaling pathway and the expression of NF-κB through the downregulation of TLR4 to promote the M2 microglia. IKK is a stimulators of NF-κB. miR-199b may downregulate IKK to inhibit NF-κB and microglia activation.

MicroRNAs are mainly transported by exosomes. Exosomes play a role in information transfer between cells by fusing with target cells and releasing the miRNAs they contain (Zhang et al., 2015). As abundantly present in the brain, miRNAs typically regulate a variety of gene expression at the translational level. In recent years, many studies have shown that miRNAs can affect the phenotype of microglia in multiple ways. It has been reported that exosomes containing miRNA-126, miR-223-3p, miR-30d-5p may inhibit M1 polarization of microglia by directly suppressing the synthesis of inflammatory factors (Jiang et al., 2018; Geng et al., 2019; Zhao Y. et al., 2020). MiR-21-5p and miR-214-5p are also thought to act on microglia, with the former promoting M1 polarization and releasing INOS from microglia, and the latter being encapsulated by GBM-derived exosomes that act directly and inhibit the activation of microglia by suppressing the relative expression of CXCL5 at the transcript and protein levels in microglia, both promoting IL-6, IL-8, Tumor necrosis factor-α(TNF -α) and other pro-inflammatory substances (Yang et al., 2019; Yin et al., 2020). NF-κB family transcription factors are typical pro-inflammatory mediators that are associated with M1 polarization in microglia (Ganbold et al., 2020). Exosomal-transported miR-124-3p inhibits microglia activation by suppressing Myosin heavy chain 9 (MYH9) expression in microglia; astrocyte-derived miR-873a-5p inhibits the NF-κB signaling pathway and thus inhibits microglia M1 polarization (Jiang et al., 2020; Long et al., 2020). In addition, IκB kinase (IKK), Nuclear Receptor 4A (Nr4a2), and toll-like receptor (TLR) were found to have possible regulatory effects on NF-κB signaling pathway. MiR-199B can downregulate IKK to inhibit NF-κB signaling pathway and thus inhibit microglial M1 polarization in microglia (Zhou et al., 2018). Nr4a2 is a suppressor of the NF-κB pathway. Exosomes of mast cell-derived miR-409-3p can promote microglia activation by downregulating Nr4a2 to promote NF-κB signaling pathway activation (Yang et al., 2019). In addition, miR-216a-5p (HExos), miR-93 may inhibit the release of inflammatory factors and M1 polarization of microglia by suppressing NF-κB signaling pathway through downregulating TLR4 expression (Liu W. et al., 2020; Wang L. et al., 2020). MiR-216a-5p also activates the phosphoinositide 3-kinase (PI3K)/AKT signaling pathway through the downregulation of TLR4 to promote the microglial M2 phenotype (Liu W. et al., 2020). In addition, TLR pathways may also directly affect microglia polarization. In contrast to miRNA-26b-5p (which inhibits M1 polarization by promoting CH25H), miR138 promotes M1 polarization by promoting TLR7/NF-κB pathway (Thomi et al., 2019; Li G. et al., 2020; Liao et al., 2020; Long et al., 2020). In addition to the above mechanisms, Bone Marrow Mesenchymal Stem Cells exosome-derived miR-146a-5p can reduce neuronal apoptosis and inhibit microglia M1 polarization by downregulating interleukin 1 receptor-associated kinase 1 (IRAK1) expression and reducing T cell 5 (NFAT5) activation (Duan et al., 2020). The current study shows that miRNAs can both inhibit microglia M1 polarization and promote M2 polarization. This could provide a new idea for the regulation of microglia polarization (Figure 1).

### LncRNA and Microglia Polarization

**FIGURE 2:**
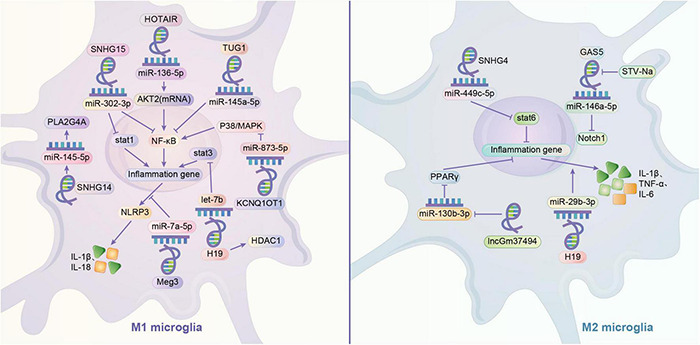
Regulation mechanism of lncRNA/miRNA axis on microglia polarization. It is very common to see that lncRNA can play a role in regulating the microglia through mediating the miRNA, like sponging the miRNA. By sponging the miR-136-5p, HOTAIR may activate M1 phenotype by upregulating AKT2 to upregulate NF-κB. Similarly, by sponging the miRNA, TUG1 may activate the microglia through NF-κB. Meg3 and sponged miR-7a-5p may together upregulate the NLRP3 to promote M1 phenotype. GAS5 may increases the expression of Notch1 protein signaling through sponging miR-146a-5p to promote M1 polarization in microglia, but this influence can be disrupted by STV-Na to convert to M2. Meanwhile, one of the crucial pathways in switching the polarization of microglia is the activation of STAT family. STAT1 and STAT3 are related to M1 phenotype, while STAT6 is related to M2 phenotype. SNHG15 may promotes STAT1 and its NF-κB expression by sponging miR-302a-3p. H19 may sponge let-7b to promote STAT3 activation, and in some cases, it might activate the M1 phenotype by increasing the HDAC1. However, H19 may also exert a promotion of M2 phenotype by sponging miR-29b-3p. Contrary to M1 phenotype, SNHG4 may promote M2 polarization in microglia and activate the STAT6 by sponging miR-449c-5p. LncRNA may downregulate the expression of miRNA. KCNQ1OT1 promotes M1 polarization in microglia by downregulating miR-873-5p to promote TRAF6 -mediated p38/MAPK pathway. SNHG14 promotes M1 phenotype by suppressing the expression of miR-145-5p on PLA2G4A. lncGm37494 promotes M2 polarization by inhibiting miR-130b-3p to promote the PPARγ pathway.

Current studies suggest that lncRNAs play important regulatory roles in gene expression and remodeling in the eukaryotic genome. LncRNAs have regulatory effects on microglia polarization in different contexts. ceRNA hypothesis suggests that multiple RNAs, including lncRNAs, can bind competitively to miRNA binding sites, curtailing the effects of miRNAs on downstream factors, which may be potential mechanism for the involvement of lncRNAs in microglial cell polarization (Thomson and Dinger, 2016). By looking at muscle-specific lncRNA lincMD and human embryonic cell lncRNA linc-RoR, it was found that lncRNA has the role of regulating multiple miRNAs and isolating miRNAs, respectively, and it may be an effective natural miRNA sponge, i.e., an efficient inhibitor of miRNAs (Webster and Thomas, 2016). Small nucleolar RNA host genes (SNHG) are a recently identified subgroup of lncRNAs that are closely associated with tumor development. SNHG4 can promote M2 polarization in microglia by sponging miR-449c-5p and thereby activating the signal transducer and activator of transcription 6 (STAT6) signaling pathway (Zhang et al., 2020). In contrast, SNHG15 promotes STAT1 and its NF-κB expression by sponging miR-302a-3p, contributing to the M1 polarization of microglia (Hu et al., 2021). In addition, the remaining sponging combinations were lncRNA TUG1/miR-145a-5p promoting M1 polarization, lncRNA HOTAIR/miR-136-5 promoting AKT2 expression and thus upregulating NF-κB for M1 polarization, lncRNA parent expression gene 3 and sponged miR-7a-5p together upregulating nod-like receptor protein 3 (NLRP3) to promote M1 polarization (Duan et al., 2018; Wang et al., 2019; Meng et al., 2021). GAS5 also increases the expression of Notch1 protein signaling through spongiotic miR-146a-5p to promote M1 polarization in microglia, but this spongiosis can be disrupted by Isosteviol Sodium (STV-Na) to convert to M2 (Zhang et al., 2019c). In addition, it was demonstrated that lncRNAs regulate microglia polarization by regulating downstream miRNAs. SNHG14 in SNHG promotes microglia M1 polarization by suppressing the expression of miR-145-5p on Phospholipase A2 (Qi et al., 2017). LncRNA KCNQ1 overlapping transcript 1 promotes microglia M1 polarization by downregulating miR-873-5p to promote TRAF6 (TNF receptor associated factor 6)-mediated p38 and NF-κB pathways (Liu N. et al., 2021). Exosomes of lncGm37494 promote microglia M2 polarization by inhibiting miR-130b-3p to promote the PPARγ pathway (Shao et al., 2020). Interestingly, different downstream miRNA axes may allow the same lncRNA to play completely opposite roles. LncRNAH19 is a classical lncRNA that can be activated by hypoxia, and H19 can bind and downregulate let-7b (a miRNA) to promote signal transducer and activator of transcription 3 (STAT3) activation in hippocampal glial cells (including microglia). Whereas exosome-derived H19 from BMSCs H19 can regulate lipopolysaccharide-stimulated microglia differentiation to M2 phenotype and attenuate neurotoxicity from inflammatory factors by inhibiting miR-29b-3p (Han C. L. et al., 2020; Zong et al., 2021). Similar to miRNAs, lncRNAs can also affect microglia polarization by regulating gene expression. H19 knockdown drives histone deacetylase inhibitor (HDAC1), which inhibits M1 microglia and exerts neuroprotective effects (Han et al., 2018b). The lncRNA HOXA-AS2 can contribute to microglia M1 polarization by interacting with the polycomb repressor complex 2 complex and downregulating proliferator-activated receptor γ coactivator (PGC-1α) (Yang et al., 2021) (Figure 2).

## Microglia Polarization and Pathogenic Mechanisms in the Central Nervous System

### Microglia Polarization and Neuroinflammation

#### Microglia Polarization and Pro/Anti-inflammatory Cytokines

Usually neuroinflammation plays a damaging role, but neuroinflammation can also play a background-dependent protective role. CNS inflammation is mainly regulated by M1/M2 microglia. Resting microglia can be activated to M1 phenotype by LPS or IFN-γ to express pro-inflammatory cytokines, or polarized to M2 phenotype by IL-4 or IL-13 to suppress inflammation and repair tissue. Aβ and Tau proteins also induce microglia to convert to pro-inflammatory phenotype (Orihuela et al., 2016). Substances such as Aβ cause microglia to produce tumor necrosis factor, IL-1β and other inflammatory cytokines by stimulating TLRs and NRLP3 inflammatory vesicles (Yang et al., 2020). These released cytokines normally protect the CNS and benefit the host, but excessive inflammatory activation can lead to pathological effects (DiSabato et al., 2016). During inflammation, over-activated microglia can produce cytokines and matrix metalloproteinases (MMPs) that further impair blood-brain barrier (BBB) function (Iadecola and Anrather, 2011). Resveratrol and PPARγ activators can promote the anti-inflammatory effect of M2 polarization by inhibiting M1 polarization and are expected to be therapeutic agents for neuroinflammation-related neurological diseases (Yang et al., 2017; Ji et al., 2018).

#### Synergistic Effects Between Microglia and Astrocytes

Similar to microglia, astrocytes can be broadly classified into A1/A2 phenotypes. Classical activation of microglia can release inflammatory mediators such as Il-1α, TNF and complement component 1q (C1q) to convert astrocytes to the neurotoxic A1 phenotype, thereby mediating the death of neurons and oligodendrocytes, among others (Liddelow et al., 2017; Kwon and Koh, 2020). In contrast, astrocytes of the A2 phenotype exert neuroprotective effects (Li T. et al., 2020). Under physiological conditions, crosstalk between astrocytes and microglia may maintain the homeostasis of the internal environment and, conversely, may lead to the development of neurological diseases (Jha et al., 2019; Liddelow et al., 2020). Manganese toxicity-induced activation of NF-κB signaling in microglia, which regulates cytokines and chemokines, promotes the A1 phenotype of astrocytes and amplifies the induced inflammatory response (Kirkley et al., 2017). Recent studies have shown that NLY01 (a novel glucagon-like peptide 1 receptor agonist) can prevent astrocyte transformation by blocking the release of inflammatory mediators from microglia (Yun et al., 2018; Hinkle et al., 2019). Notably, astrocytes can also inhibit microglia activation and reduce inflammation by expressing orosomucoid2 that binds to microglia C-C chemokine receptor type 5 (CCR5), thereby blocking the interaction of CCL4 and CCR5 that chemotactic neutrophils infiltrate across the BBB to sites of inflammation (Jha et al., 2019). In addition, astrocyte-specific glutathione S-transferase M1 activates pro-inflammatory signaling regardless of *in vivo* and *in vitro* culture, thereby enhancing microglia-activated inflammatory responses (Kano et al., 2019).

### Link Between Microglia Polarization and Oxidative Stress

Oxidative stress associated with microglia polarization is also a pathogenic mechanism in many CNS diseases. The active metabolite of VitD, ossified triol, can prevent oxidative stress induced by traumatic brain injury (TBI) by inhibiting microglia M1 polarization and enhancing M2 polarization (Cui et al., 2019). Microglia themselves are also an important source of oxidative stress, and activation of their NADPH oxidase 2 (NOX2) enzyme contributes to the production of superoxide compounds (Yauger et al., 2019). Sodium fluoride induces oxidative stress in microglia and promotes the release of pro-inflammatory factors by activating the NF-kB pathway. This process can be reversed by enhanced nuclear factor erythroid 2-related factor 2 (Nrf2) (Song et al., 2020). In TBI, iron promotes Reactive Oxygen Species (ROS) synthesis by microglia and promotes microglia-associated oxidative stress without altering microglia polarization (Yauger et al., 2019). Several studies have shown that NOX2 and non-specific NOX inhibitors and iron chelation therapy reduce inflammation after injury (Wang et al., 2014; Li et al., 2016). In addition, Chronic social defeat is also associated with ROS production after microglia activation (Lehmann et al., 2019). In addition to their own ROS release leading to oxidative stress, M1 microglia inducing neuroinflammatory damage can also lead to ROS production in their peripheral nerve cells, thus further amplifying oxidative stress injury. Hydrogen peroxide acting on microglia will contribute to microglia activation and proliferation (Mander et al., 2006).

### Microglia Polarization and Programmed Neuronal Cell Death

#### Microglial Pyroptosis: Inflammatory Cell Death of Pro-inflammatory Microglia

A large number of recent studies have elucidated the link between microglia pyroptosis and multiple CNS diseases. Inflammatory vesicle activation is known to be associated with the development of MS, AD, TBI, and many other neurological disorders (Freeman and Ting, 2016; McKenzie et al., 2020). Inflammatory vesicles are important influences to initiate pyroptosis and inflammation. It has been demonstrated that inflammatory vesicles activate in microglia under ischemic conditions, activating caspase-1 and thus inducing microglia focal death (Poh et al., 2019). Microglia focal death may be an important mechanism for inflammatory vesicle-mediated further progression of various brain diseases. In a rat model, NLRP3 and NLRP4 inflammatory vesicles mediate microglia pyroptosis-induced ischemic-hypoxic brain injury (Poh et al., 2019; Chang et al., 2020). Inflammatory vesicles such as NLRP3 are the crossover hub of inflammatory and pyroptosis pathways. Due to the interference of neuroinflammation, the above demonstration cannot directly prove the involvement of microglia pyroptosis in the pathogenesis of various neurological diseases. Recent studies have reported that in MS models, inhibition of caspase-1 can reduce microglia pyroptosis and thus exert neuroprotective effects (McKenzie et al., 2018; Poh et al., 2019). It has also been shown that knockdown of Gasdermin-D (GSDMD) or inhibition of GSDMD with GSDMD-C, afenide, etc. can effectively reduce the secretion of mature IL-1β and IL-18 and microglia pyroptosis and subsequent ischemic stroke-related brain injury (Zhang et al., 2019b; Han C. et al., 2020; Wang K. et al., 2020). Celastrol may alleviate spinal cord inflammation in rats in the acute phase by inhibiting microglia pyroptosis (Dai et al., 2019). c-Jun N-terminal kinases also reduce the time to neurocognitive recovery after surgery by inhibiting microglia pyroptosis (He et al., 2021). In addition, lncRNA F630028O10Rik, lncRNA H19, and CD73 are involved in the progression of neurological diseases by promoting microglia pyroptosis (Wan et al., 2020; Xu et al., 2020a,^2021^). It has been mentioned previously that NF-κB activation will promote microglia M1 polarization. In addition, NF-κB is also able to promote the expression of NLRP3. Under certain conditions of stimulation such as Aβ, NLRP3 activation will be promoted and focal death will be initiated (Yang et al., 2020). Thus, M1 microglia have a higher susceptibility to cellular pyroptosis compared to M2 type. This suggests that inhibition of microglia M1 polarization may avoid the development of neuroinflammation brought about by pyroptosis.

#### Pro-inflammatory/Anti-inflammatory Ratio Imbalance and Iron Death

Currently, a strong link between iron death and neurodegeneration has been found in experimental animal models of various neurological diseases (Stockwell et al., 2017; Weiland et al., 2019; Qiu et al., 2020). However, only a few studies have provided direct evidence. Anti-iron death is thought to have a possible protective effect on neurons (Mahoney-Sánchez et al., 2021). In a middle cerebral artery occlusion (MCAO) model, inhibition of iron death attenuated neuronal injury after ischemia in mice, and iron chelation also reduced ischemia-reperfusion injury (Prass et al., 2002; Tuo et al., 2017). Deficiency of the neuronal iron transporter protein ferroportin (Fpn) can promote iron death (Bao et al., 2021). And in a mouse spinal cord injury model, expression of both pro-inflammatory cytokine TNF-α and anti-inflammatory cytokine TGF-β1 under inflammatory conditions caused inhibition of FPN expression in microglia (Rathore et al., 2012). In aged mice exposed to acute inflammatory injury, overexpression of heme oxygenase-1 Heme oxygenase-1 (HO-1) in microglia contributed to iron accumulation and iron death, whereas knockdown and inhibition of HO-1 significantly ameliorated these phenomena (Fernández-Mendívil et al., 2021). A potential link between microglia-induced pro- and anti-inflammatory activities and iron death is implied. As mentioned above, M2-type microglia inhibit inflammation and iron death-dependent oxidative stress and may exert an inhibitory effect on iron death, whereas M1-type acts in the opposite direction. However, these need further studies to be demonstrated. In addition, polarization of microglia is also thought to be associated with resistance to iron death. Different phenotypes of microglia differ in their susceptibility to iron death. M1-polarized microglia exhibit higher resistance, whereas M2-polarized microglia exhibit higher sensitivity, which may be related to the elevated M1/M2 ratio under oxidative stress conditions (Kapralov et al., 2020). Further studies on the link between microglia polarization and iron death are warranted.

#### Microglia Polarization Regulates Apoptosis

Activated M1 microglia can contribute to neuronal apoptosis by mediating immune inflammation occurring, and their inflammatory mediators may be responsible for apoptosis. It has been reported that neuroapoptosis in Toxoplasmic encephalitis may not be parasitic in origin, but may contribute to neuronal apoptosis by indirectly activating microglia to release large amounts of inflammatory factors (Zhang et al., 2014). In schizophrenic patients, abnormal dendritic spine pruning mechanisms are thought to be associated with neuronal apoptosis (Fricker et al., 2018; Parellada and Gassó, 2021). Activated microglia contribute to the abnormal reduction of dendritic spine density in schizophrenic patients, and the use of the antibiotic dimethylamine-tetracycline reduces microglia-mediated synaptic pruning (Parellada and Gassó, 2021). In addition, sustained or excessive microglia activation leads to the release of many cytotoxic and inflammatory factors that induce apoptosis, such as neuronal apoptosis induced by sustained microglia activation due to overexposure to fluoride (Yan et al., 2016).

In the past, it was thought that over-activated M1 microglia might activate their own apoptosis through self-regulation to stop the continued expansion of inflammation. Among them, TLR4 and IFN-β seem to play an important role, but the exact molecular mechanism is still not elucidated (Liu et al., 2001; Yang et al., 2002; Jung et al., 2005; Ge et al., 2016). Thus, apoptosis or depletion of activated microglia is thought to be an important mechanism for impeding the progression of neurological diseases. The antidepressants fluoxetine and desmethyl fluoxetine can induce apoptosis of activated microglia (Dhami et al., 2019). Activated M1 microglia have also been found to have inhibitory effects on synaptic recovery and neurite growth, and studies have shown that depletion of such activated microglia with PLX3397 reduces dendritic spine loss and neuronal apoptosis in moderate TBI (Wang et al., 2019; Wang C. F. et al., 2020). Therefore, inhibition of microglia activation and induction of apoptosis of activated microglia may reduce the neurological damage they cause.

### Microglia Polarization and Synaptic Plasticity

It has been shown that disruption of interactions between microglia and neurons leads to an increase in immature synaptic connections. Microglia selectively phagocytose synapses and induce postsynaptic spine head filopodia (Weinhard et al., 2018). These suggest that microglia can promote synaptic remodeling and circuit maturation. Chronic depletion of microglia in adult visual cortex has been reported to enhance local neural circuit connectivity (Liu Y. J. et al., 2021). Microglia may influence synaptic plasticity through multiple pathways, including phagocytosis, inflammatory factors, immunomodulatory factors, and coactivation with other neurons. During normal brain development, microglia maintain proper neuronal connections through synaptic pruning, thereby establishing stable and mature CNS circuits (Paolicelli et al., 2011; Wu et al., 2015). Long-term potentiation (LTP) and long-term depression (LTD) are indicators of the strength of synaptic connections and are thought to be the molecular basis of synaptic plasticity in learning and memory (Diering and Huganir, 2018). In studies of major depressive disorder, chronic stimulation in inflammation may lead to the release of inflammatory mediators IL-1β and TNF-α from microglia, which can impair the induction of LTP by preventing phosphorylation of the alpha-amino-3-hydroxy-5-methyl-4-isoxazolepropionic acid (AMPA) receptor GluR1 subunit (which can impair the induction of LTP). Preventing AMPA receptor GluR1 subunit phosphorylation) (Innes et al., 2019). IL-1β and its receptors are mainly expressed in the hippocampus, and abnormally elevated concentrations have been shown to reduce LTD in certain regions, thereby impairing synaptic plasticity (Innes et al., 2019). BDNF is a neurotrophic factor secreted by microglia that promotes neuronal survival and directly affects the structure and function of nearby synapses, and its reduction reduces activation and phosphorylation of synaptic TrkB receptors, inhibits the expression of glutamate transporters and N-methyl-D-aspartate receptors, and ultimately impairs LTP (Parkhurst et al., 2013). While BDNF levels in microglia decrease as mice age, local supplementation of BDNF can inhibit microglia activation (Wu et al., 2020). Microglia may also be able to mediate synaptic plasticity through interactions between the chemokine receptor C-X3-C motif chemokine receptor 1 (CX3CR1) and other neuronal CX3C chemokine ligand 1 (CX3CL1) ligands (Ragozzino et al., 2006; Bolós et al., 2018). Both a decrease in CX3CL1 and a decrease in CX3CR1 receptors on microglia usually lead to microglia activation (Mecca et al., 2018; Zhang J. et al., 2018). In particular, decreasing CX3CL1 and increasing CX3CR1 were found in TG mice during ALS progression late in the disease, and this imbalanced CX3CL1/CX3CR1 axis seems to be closely related to the microglia M1/M2 imbalance (Zhang J. et al., 2018). In addition, the extracellular matrix network may provide protection to neurons to limit their plasticity, and microglia may be instructed by neuronal IL-33 to remodel the extracellular matrix (Nguyen et al., 2020; Zaki and Cai, 2020). Meanwhile, IL-33 is a new regulator of microglia activation and function produced by astrocytes (Vainchtein et al., 2018).

### Pro-inflammatory/Anti-inflammatory Microglia and Blood-Brain Barrier Disruption and Protection

The integrity of the BBB is critical to the health of the brain. Both aging and inflammatory responses can cause disruption of the BBB, leading to the entry of harmful substances into the brain (Sweeney et al., 2018). Endothelial dysfunction is an important cause of BBB damage, and BBB damage caused by aging may be alleviated by inhibiting endothelial cell C3a receptors (Propson et al., 2021). Tight Junctions and Adherens Junctions are major proteins that control vascular permeability, and disruption of both protein complexes, especially the adherens junctions complex, leads to altered cellular junctions, which are found in a variety of neurodegenerative neurological diseases (Maiuolo et al., 2018). In addition, the expression of occludins and Zona occludens 1 (ZO-1) correlates with BBB permeability (Miyazaki et al., 2011).

**FIGURE 3:**
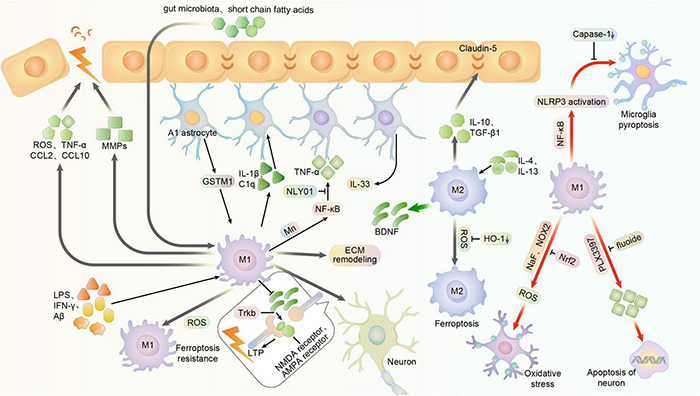
Multiple function of microglia polarization in the central nervous system. Usually, microglia can be activated to M1 phenotype in central system by LPS, IFN-γ, and Aβ, exerting pro-inflammatory effects. And it can be stimulated to M2 phenotype by IL-4 and IL-13, exerting anti-inflammatory effects. NaF and NOX2 may promote the oxidative stress of microglia and its subsequent impact, while it can reversed by enhanced Nrf2. Meanwhile, the substances like Short-chain fatty acids and gut microbiota that are outside the BBB can also activate microglia by crossing the BBB. However, activated M1 microglia can damage the blood-brain barrier by secreting ROS, TNF-α, chemotactic proteins CCL2 and CXCL10. And in some cases, the over-activated microglia may produce cytokines and MMPs that further impair blood-brain barrier function. The integrity of BBB is related to the cellular junctions, in which the claudin-5 is an important factor. And the M2 phenotype of microglia can produce IL-10 and TGF-β1 to stabilize the BBB. Astrocyte-specific GSTM1 may enhance inflammatory responses of activated microglia. And microglia can release inflammatory mediators such as Il-1α, TNF and C1q to convert astrocytes to the neurotoxic A1 phenotype, enhancing the inflammation. Mn toxicity-induced activation of NF-κB signaling in microglia, which can also regulate cytokines and chemokines, promotes the A1 phenotype of astrocytes. NLY01 can prevent the microglia from releasing these mediators and cytokines, thus inhibiting the conversion of astrocyte. Astrocyte can also produce IL-33, which might activate microglia to remodel the extracellular matrix, showing a capability to restrain the synaptic plasticity. M1 microglia can prevent the BDNF from releasing, thus reducing activation and phosphorylation of synaptic TrkB receptors and inhibiting the expression of glutamate transporters and NMDA receptors, ultimately impairs LTP. But in other cases, M2 microglia might produce BDNF to improve it. The fluoxetine can induce apoptosis of activated microglia, while depletion of such activated microglia with PLX3397 reduces neuronal apoptosis. NF-κB is able to promote the activation of microglia and the expression of NLRP3, thus initiating pyroptosis of microglia. But the inhibition of caspase-1 reduces microglia pyroptosis. M1-polarized microglia exhibit higher resistance, whereas M2-polarized microglia exhibit higher sensitivity. But the inhibition of HO-1 may reduce the risk of ferroptosis.

The activity of microglia has an important impact on the BBB. In patients with chronic BBB dysfunction, many microglia, monocytes, and neuroinflammatory markers are present around the lesion (van Vliet et al., 2020). The current study shows that M1 microglia usually cause BBB dysfunction, while M2 type usually plays a protective role on the BBB (Ronaldson and Davis, 2020). Hormones such as leptin or short-chain fatty acids, a release product of the gut microbiota of PD patients, can cross the BBB and increase microglia activation (Sampson et al., 2016). Activated M1 microglia can damage the BBB by secreting ROS, TNF-α, chemotactic protein C-C motif chemokine ligand 2 (CCL2) and Chemokine C-X-C ligand 10 (CXCL10) to mediate inflammatory damage and immune responses (Stamatovic et al., 2005; Broux et al., 2015). It was shown that intracerebroventricular injection of brain-reconstituted CCL2 in mice resulted in reduced immunostaining of BBB-linked proteins such as occludin, claudin-5, ZO-1 and ZO-2. In contrast, IL-10 secreted by M2-type microglia has an important role in stabilizing the BBB. Both *in vivo* and *in vitro*, IL-10 reduced the downregulation of claudin-5 and damage to tight junctions thereby mitigating BBB disruption (Lin et al., 2018). In addition, microglia can also secrete TGF-β1 and attenuate BBB disruption. In contrast, excessive activation of transforming growth factor-β (TGF-β) in astrocytes can induce further neurological deficits (Diniz et al., 2019; Senatorov et al., 2019). In addition to inflammation, the hypoxic environment may also affect the BBB. It was recently found that microglia depletion in hypoxic conditions increases vascular leak in the spinal cord (Halder and Milner, 2020). This suggests that microglia are likely to play a protective role in the BBB of the brain, the exact molecular mechanism of which is unknown (Figure 3).

## Current Status of Research on the Relationship Between LncRNA, Microglia Polarization and Neurological Disorders

### Neuropathic Pain

Neuropathic pain is a common chronic pain disorder that occurs after nerve injury and can be induced by a variety of diseases such as MS, PD, spinal cord injury, stroke, and epilepsy. The close relationship between microglia activation and neuropathic pain has been well described, and their pro-inflammatory M1 and anti-inflammatory M2 phenotypes may play an important regulatory role in neuropathic pain (Inoue and Tsuda, 2018). LncRNAs, as damaging mutational regulators of gene expression, are also key regulators of neuronal function and have recently been suggested to be involved in the development of neuropathic pain. Because recent evidence suggests that abnormal lncRNA expression in damaged nerves after peripheral nerve injury, dorsal root ganglion or dorsal horn of the spinal cord may be responsible for the pathogenesis of neuropathic pain (Wu et al., 2019). LncRNAs such as KCNA2-AS, uc.48+, NON-RATT021972, MRAK009713, BC168687, XIST, CCAT1, and NEAT1 have been found to be associated with many types of neuropathic pain, including those of diabetic origin (Li Z. et al., 2019). In a rat model of chronic compression injury of the sciatic nerve (CCI), downregulation of XIST in microglia slowed neuropathic pain by reducing the expression of pro-inflammatory factors in rats with M1. Currently, XIST can act through multiple target genes by sponging miR-544 to promote STAT3 activation in microglia, sponging miR150 to upregulate Zinc finger E-box binding homeobox 1, or negatively regulating miR-154-5p and thus increasing TLR5 expression, all three pathways promote neuropathic pain development (Jin et al., 2018; Wei et al., 2018; Yan et al., 2018). Also, in a rat model of bilateral chronic constriction injury, STAT3, a transcription factor responsible for the production of pro-inflammatory cytokines and a key factor in the development of neuropathic pain, is involved in linc00311 and AK141205-induced neuropathic pain (Jin et al., 2018; Ding et al., 2019). LncRNA Colorectal tumors differentially expressing lncRNAs that also sponge miR-136 positively regulate IL-6R in neuropathic pain and increase the production of pro-inflammatory markers in microglia M1 (Zhang et al., 2019a). In addition, a novel lncRNA embryonic stem cells expressed 1 (lncenc1), which promotes neuropathic pain by interacting with EZH2 to downregulate brain-specific angiogenesis inhibitor 1 (BAI1) expression (Zhang et al., 2021). Both EZH2 expression and BAI1 downregulation are important factors in microglia activation and reversing them or directly knocking down lncenc1 may be a target to slow down disease progression (Zhang et al., 2021). In studying how guanosine-5′-triphosphate cyclohydrolase 1 mediates microglia activation involved in neuropathic pain, differential expression between multiple lncRNAs and mRNAs in microglia has attracted significant attention and is likely to be a potential and important mechanism (Liang et al., 2021).

### Epilepsy

Epileptogenesis is the process by which epileptic activity in the brain goes from absent to capable of producing spontaneous, recurrent epilepsy, and the process is thought to be the result of an imbalance of excitatory and inhibitory activity within the neuronal network (Pitkänen and Engel, 2014). Epilepsy rarely exists alone, and more than half of patients with epilepsy have comorbidities such as psychiatric disorders (e.g., depression, anxiety, autism spectrum disorders, etc.). Epigenetic mechanisms may be involved in the development of epilepsy, with epigenetic changes driving a number of related biological processes, including accumulation of neurodegenerative proteins, pro-inflammatory responses, disruption of the BBB, and proliferation or activation of microglia (Pitkänen, 2002; Hauser et al., 2018; Patel et al., 2019). In contrast to the extensive research on miRNAs in epilepsy, lncRNAs are still being enriched and are tentatively considered to have a regulatory role in epigenetic epilepsy. As post-transcriptional regulators and regulators of inflammatory and neuronal differentiation pathways in the brain, the regulation of neuroexcitability by methylation of lncRNAs and their genes may be relevant to the development of epilepsy (Hauser et al., 2018). H19 is an apoptosis- and tumor-associated lncRNA that has recently been shown to be highly expressed in the latency of epilepsy. In a rat model of temporal lobe epilepsy, lncRNAH19 is the most clearly differentiated lncRNA that can play a role in promoting apoptosis of hippocampal neurons by sponging let-7b (Han et al., 2018b). And the study specifically reported that H19 promotes Stat3 through let-7b, thus inhibiting hippocampal microglia activation and seizures (Han C. L. et al., 2020). H19 overexpression increased Janus kinase and activator of transcription (STAT), promoting microglia activation after status epilepticus (Han et al., 2018a). In addition, a lncRNAPeg13 binding to miR-490-3p drives upregulation of proteasome non-ATPase regulatory subunit 11 levels thereby inhibiting microglia activation and reducing inflammatory factor levels in the epileptic hippocampus (Feng et al., 2020). In patients with epilepsy, microglia activation may be an important influence on disease progression. There is also a large body of literature on the regulation of lncRNAs and miRNAs and their downstream pathways in epilepsy. MMP is associated with disruption of the BBB in epilepsy. lncRNAILF3-AS1 promotes the expression of inflammatory cytokines and MMP by repressing miR-212 (Cai et al., 2020). lncRNA NEAT1 upregulates Notch signaling and other M1 pro-inflammatory substances in epileptic cell models by repressing miR-129-5p (Wan and Yang, 2020). In a rat model of trichothecene induced epilepsy, lncRNAUCA1 increased Nrf2 protein levels by negatively regulating miR-495, thereby suppressing brain epilepsy (Geng et al., 2018). Similar to UCA1, the lncRNA FTX/miR-21-5p/SOX7 pathway also plays a role in suppressing epilepsy (Li X. et al., 2019).

### Ischemic Stroke

Ischemic stroke and ischemia-reperfusion are both extremely threatening injuries that can occur after ischemia and hypoxia. Ischemic stroke causes biological responses such as neuroinflammation, oxidative stress, excitotoxicity and apoptosis, which lead to neuronal death (Chen et al., 2017). In particular, activation of neuroinflammation is involved in all stages of ischemic stroke development, from injury to repair to vascular regeneration. Numerous studies have demonstrated that post-ischemic microglia activation induces NF-κB-related inflammation, which is considered as a potential mechanism of the disease (Chen et al., 2017). The study of lncRNA has also made great progress in ischemic stroke. In ischemic stroke, there is a time-dependent expression profile and significant changes in lncRNA expression levels in the brain and blood, suggesting that lncRNAs may serve as therapeutic targets or prognostic markers (Chen et al., 2019). The role of NF-κB pathway in ischemic stroke has been well described. In a MCAO and *in vitro* oxygenated glucose deprivation/reoxygenation-induced hypoxia/ischemia, SNHG15 sponges miR-302a-3p thereby promoting STAT1 and NF-κB expression and activating microglia involved in the development of ischemic brain injury (Hu et al., 2021). In *ex vivo* ischemic stroke experiments, lncRNA-1810034E14Rik may exert anti-inflammatory effects by inhibiting the NF-κB pathway and suppressing microglia activation and p65 hyperphosphorylation, with implications for stroke treatment (Zhang et al., 2019e). In addition, lncRNA RMST activated tank1-mediated NF-κB signaling pathway and promoted microglia activation through competitive binding with hnRNPK (Sun et al., 2019). The histone deacetylase histone deacetylase (HDAC) regulates inflammatory mediator production and its inhibitor promotes microglia conversion to the M2 phenotype thereby reducing neuroinflammation (Jaworska et al., 2017; Patnala et al., 2017). In ischemia-reperfused mice, lncRNASNHG3 inhibits microglia activation and secretion of pro-inflammatory factors (TNF-α and IL-6) by decreasing histone deacetylase 3 (HDAC3) expression (Huang et al., 2021). H19 is a lncRNA that can be activated by hypoxia and is significantly upregulated in a rat model of cerebral ischemia-reperfusion. In a mouse model of ischemia, downregulation of H19 may reduce ischemic injury by inhibiting HDAC1 expression and thereby polarizing microglia to M2 type (Wang et al., 2017). LncRNANEAT1 is also significantly expressed in ischemic stroke patients, and according to genome-wide RNA-seq studies, it may promote microglia activation (Jin et al., 2021). Knockdown of lncRNA nuclear-enriched abundant transcript 1 (NEAT1) reduces the activity of the proinflammatory AKT/STAT3 pathway and inhibits microglia activation, reducing ischemia-reperfusion-induced injury (Ni et al., 2020).

### Neurodegenerative Diseases

As the population ages, the prevalence of neurodegenerative diseases, including Alzheimer’s, Parkinson’s, and HD, has increased. The main pathological manifestations of neurodegenerative diseases are progressive neurodegeneration and protein amyloid deposition or even pathological proliferation (amyloid includes Aβ, tau proteins and α-systemic nucleoproteins), and these diseases are thought to have a broad relevance to mammalian prions (Heemels, 2016; Zhu et al., 2016). In neurodegenerative diseases, disease progression is closely linked to microglia activation and systemic inflammation, and the development of AD revolves around Aβ plaques, hyperphosphorylated tau proteins, and microglia, with Aβ and its proinflammatory factors contributing to decreased microglia clearance, and later microglia more likely to interact with Aβ leading to NLRP3 inflammatory vesicle activation, neuronal loss etc. (Hickman et al., 2018). Of note, the acute and chronic activation of microglia has implications for guiding the treatment of disease. Microglia are usually activated chronically in Alzheimer’s disease, Parkinson’s disease, and neuropathic pain. In contrast, it is activated acutely in brain hemorrhage or stroke and dynamically differentiates into typical M1 and M2 types as the disease progresses. For acute stimulation, changing the phenotype at the right time is instructive for the treatment of the disease (Lan et al., 2017). Comparatively, chronically activated microglia may worsen the condition by releasing excessive amounts of these cytotoxic factors. For example, Aβ deposition in AD and pro-inflammatory cytokines secreted by microglia further reduce microglia clearance, thus amplifying the neuronal damage caused by these toxic factors (Hickman et al., 2018). Also, activation of innate immune signaling in neurodegenerative conditions can affect microglia clearance. Therefore, the growth factor TGF-β, the complement cascade and the extracellular receptor TREM2 are of interest in the immune-related therapeutic role in chronically activated microglia-related diseases (Hammond et al., 2019). Recently, microglia associated with Aβ plaques were found to exhibit a neurodegenerative microglia phenotype, and inhibition of the APOE pathway by TREM2 ameliorated the disease by maintaining microglia homeostasis (Krasemann et al., 2017). The progression of PD may be similar to that of AD, but another subject may be α-glycosylated nucleoprotein. In addition, the important role of lncRNA in brain development, neuronal function and maintenance gives it an important impact in neurodegenerative diseases. In a PD mouse model, lncRNA-UCA1 exacerbates the development of PD by upregulating α-glycosylated nucleoprotein (Lu et al., 2018). In mice or BH-SY5Y microglia induced *in vivo* and *in vitro* PD models, it was found that inhibition of lncHOXA11-AS inhibited neuroinflammation by upregulating miR-124-3p-follistatin-like 1 by suppressing the NF-κB pathway, and both of these ncRNA alterations significantly suppressed microglial cell activation and thus attenuated the development of PD (Cao et al., 2021). In addition, microglia activation and extensive increase in pyrin structural domain 3 (NLRP3) inflammatory vesicles were observed in brains collected from PD patients, suggesting that microglia NLRP3 inflammatory vesicle activation may be a potential target for treatment (Haque et al., 2020). In mouse models of PD and LPS-activated microglia, lncGAS5 was found to promote NLRP3 expression and thus accelerate disease progression by sponging miR-223-3p (Xu et al., 2020b). In addition, the development of AD is thought to be associated with microglia activation in addition to many miRNAs with lncRNAs (Zhao et al., 2019; Ellwanger et al., 2021). In the well-characterized PD25 acute MPTP mouse cell model, lincRNA-p21 can induce microglia activation by competitively binding to the miR-181 family and thus promoting the Protein kinase C (PKC-δ) pathway. At the same time, PKC-δ can also contribute to p53/lincRNA-p21 expression, thus forming a circuit that continuously enables microglia activation and neurodegenerative disease progression (Ye et al., 2018). The accumulation of LINC-PINT in response to oxidative stress was recently identified in PD and AD patients and may decrease with aging, suggesting a possible neuroprotective role of this lncRNA in neurodegenerative diseases (Simchovitz et al., 2020). There is still a lack of research on microglia and lncRNA in AD. The function of microglia in AD is very complex, and the diversity of microglia phenotypes in AD has been identified by transcriptome and meta-analysis. The phenotypes of microglia associated with AD pathogenesis described previously, namely the MGnD and MG-dNF phenotypes, are also known as phagocytosis-associated disease-associated microglia (DAM) phenotypes. TREM2, a ligand for Aβ, has been shown to be expressed in correlation with phagocytosis in AD, both *in vivo* and *in vitro* experiments (Qin et al., 2021). It is suggested that TREM2 expression correlates with microglia survival, promotes the transition of steady-state microglia to the DAM state, and serves to regulate microglia proliferation and effector functions (Keren-Shaul et al., 2017). The specific relationship between TREM-related phenotypes of microglia and lncRNAs deserves further research exploration.

### Depression

Traditional antidepressants and electroconvulsive therapy have improved the structure and function of microglia to some extent, suggesting that targeting microglia may be one of the strategies for depression treatment (Yirmiya et al., 2015). In animal models, lipopolysaccharide-induced activation of microglia plays an important role in the pathogenesis of depression (Yirmiya et al., 2001). Studies suggest that by regulating microglia polarization may be the mechanism of action of many antidepressants (Xu et al., 2018). It has been recently suggested that the severity of symptoms in some major depressive disorders correlates with elevated levels of inflammatory factors in plasma, most likely due to microglia activation induced by certain pathogens with high affinity for the brain (Yirmiya et al., 2015). In addition, a meta-analysis of 36 studies found that almost all of these anti-inflammatory drugs had an ameliorating effect on depression (Köhler-Forsberg et al., 2019). The close association of lncRNAs with synaptic plasticity, cognitive function, and microglia regulation suggests that they may regulate major depressive disorder, while genome-wide analysis revealed multiple lncRNAs differentially expressed in major depressive disorder patients (e.g., LINC00473), four of which may have synergistic effects with the corresponding four mRNAs in regulating major depressive disorder development (Liu et al., 2014). It was found that CD206, a typical marker of microglia M2 polarization, was significantly downregulated in patients with bipolar disorder (Ohgidani et al., 2016). Currently, lncRNA uc.80-downregulation, M1 polarization of microglia and downregulation of CD206 were found in the hippocampus of depressed patients and rats. It was found that high expression of lncRNA uc.80- in mice significantly upregulated CD206 expression and promoted the conversion of microglia from M1 to M2, thereby and improving depression (Gu et al., 2020). In addition, lncRNA uc.80- together with many other lncRNAs (XR_009031, XR_9360, MRAK035378, uc.123+, XR_008644 and AJ535460) and their cis/trans regulatory target genes (Zbtb20, Zfp385b, Gemin6, Kif2) may be involved in fluoxetine in the antidepressant effect of fluoxetine (Liu N. et al., 2020). Notably, the overall behavioral changes may be the result of the cumulative action of multiple lncRNAs, and more research is needed regarding the regulatory role of lncRNAs in depression. Current research on the role played by lncRNAs targeting microglia polarization in depression is still very limited.

### Other Neurological Disorders

In addition to the important regulatory role played by lncRNAs in the above neurological disorders, related studies suggest a link between lncRNAs and many other neurological disorders. LncRNAs such as DISC-2, Gomaf, and Evf2 have also been suggested to be involved in the regulation of schizophrenia (Merelo et al., 2015). The motor neuron diseases amyotrophic lateral sclerosis and spinal muscular atrophy have also recently been suggested to be associated with lncRNA regulation, and targeting MN markers by lncRNAs may be a new therapeutic option for them (Chen and Chen, 2020). The application of lncRNAs in neurological diseases has a good prospect, and the association between different lncRNAs and different neurological diseases deserves further research and development (Table 1).

**TABLE 1:** Effects of lncRNA on neurological diseases by regulating microglia polarization.

LncRNA	Diseases and models	Mechanism	Microglia phenotype	Outcomes	References
XIST knockdown	Neuropathic pain (*in vivo*)	Decreasing XIST and pro-inflammatory factors	M2	Decreasing the progression of the disease	Yan et al., 2018
CRNDE	Neuropathic pain (*in vivo*)	Decreasing miR-136 ang raising IL-6R and pro-inflammatory factors	M1	Promoting neuropathic pain progression	Zhang et al., 2019a
Lncenc1	Neuropathic pain (*in vivo* and in *in vitro*)	Interacting with EZH2 and decreasing Bai1	M1	Leading to neuropathic pain	Zhang et al., 2021
H19	Epilepsy (*in vivo*)	Raising H19, STAT3 and decreasing let-7b	M1	Promoting epileptic seizure	Han C. L. et al., 2020
H19	Epilepsy (*in vivo*)	Raising H19, JAK, STAT	M1	Promoting epileptic seizure	Han et al., 2018a
LncRNAPeg13	Epilepsy (*in vivo*)	Binding to miR-490-3p and decreasing Psmd11 and inflammatory factor	M2	Decreasing the level of the inflammatory factors	Feng et al., 2020
SNHG15	Ischemic stroke (*in vivo* and in *in vitro*)	Decreasing miR-302a-3p and rising STAT1 and NF-κB expression	M1	Ischemic brain injury	Hu et al., 2021
1810034E14Rik	Ischemic stroke (*in vivo* and in *in vitro*)	Decreasing NF-κB pathway and p65 hyperphosphorylation	M2	Decreasing the infarct volume Alleviating brain damage in MCAO mice	Zhang et al., 2019e
RMST	Ischemic stroke (*in vitro*)	Competitively binding to hnRNPK and rising tank1-mediated NF-κB pathway	M1	Ischemic brain injury	Sun et al., 2019
SNHG3	Ischemic stroke (*in vitro*)	Decreasing pro-inflammatory factors and HDAC3	M2	Reducing ischemic injury	Huang et al., 2021
H19 knockdown	Ischemic stroke (*in vivo* and *in vitro*)	Decreasing H19 and HDAC1	M2	Reducing ischemic injury	Wang et al., 2017
LncNEAT1 knockdown	Ischemic stroke (*in vivo* and *in vitro*)	Decreasing NEAT1 and proinflammatory AKT/STAT3 pathway	M2	Reducing ischemia-reperfusion-induced injury	Ni et al., 2020
lncHOXA11-AS knockdown	Parkinson’s disease (*in vivo* and *in vitro*)	Decreasing miR-124-3p and the pathway of FSTL1 and NF-κB	M2	Decreasing the progression of Parkinson’s disease	Cao et al., 2021
lncGAS5	Parkinson’s disease (*in vivo* and *in vitro*)	Rising NLRP3 and decreasing miR-223-3p	M1	Accelerating disease progression	Xu et al., 2020b
lincRNA-p21	Parkinson’s disease (*in vitro*)	Competitively binding to the miR-181 and rising PKC-δ pathway	M1	Forming a circuit that continuously enables microglia activation and neurodegenerative disease progression	Ye et al., 2018
lncRNA uc.80-	Depression (*in vivo* and *in vitro*)	Decreasing CD206 expression	M2	Improving depression	Ohgidani et al., 2016

*EZH2, Enhancer of zeste homolog 2; Bai1, Brain-specific angiogenesis inhibitor 1; STAT3, signal transducer and activator of transcription 3; JAK, Janus kinase; Psmd11, proteasome non-ATPase regulatory subunit 11; hnRNPK, Heterogeneous nuclear ribonucleoprotein K; HDAC3, histone deacetylase 3, histone deacetylase 3; HDAC1, histone deacetylase 1; NEAT1, nuclear-enriched abundant transcript 1; FSTL1, follistatin-like 1; PKC-δ, Protein kinase C-δ; MCAO, middle cerebral artery occlusion.*

## Summary and Outlook

Different types of lncRNAs regulate signaling pathways associated with microglia polarization by regulating the expression of miRNAs or genes. Microglia play a key role in the regulation of CNS diseases. Microglia polarization is currently a hot topic of research and is expected to be widely used in the clinical treatment of CNS diseases. Curcumin, candesartan and resveratrol all inhibit the NF-κB pathway thereby suppressing M1 polarization and promoting microglia M2 polarization, and all of these drugs reduce neuroinflammatory damage. In addition, there is the commonly used PPAR-γ agonist rosiglitazone that promotes microglia M2 polarization. The therapeutic effects of these drugs on neurological disorders are in the exploratory stage. Therefore, more drugs targeting microglia may be put into research and use in the future.

Under normal conditions, microglia maintain the homeostasis of the brain’s internal environment by regulating neuroinflammation, oxidative stress, programmed cell death, synaptic plasticity and BBB integrity. Generally speaking, M1 microglia promote neuroinflammation, oxidative stress, microglial cell death, neuronal apoptosis, synaptic plasticity damage and BBB disruption. And in contrast, M2 microglia have the opposite effect. Furthermore, M1-type microglia have a stronger resistance to iron death in the context of oxidative stress relative to M2-type, which would further lead to an increase in the M1/M2 ratio, thereby exacerbating the neurological disorder. However, the reasons for the stronger resistance to oxidative stress and iron death acquired by M1-type microglia deserve further research to explore.

Many drugs currently show the property of reducing damage by inducing their conversion to M2-type cells *in vitro*, however, the regulatory mechanisms are often more complex. In recent years, epigenetic studies have emerged and a large body of literature suggests that lncRNAs and miRNAs have an important role in treating neurological diseases by targeting microglia behavior. In particular, the nanotechnology has made it possible for humans to carry drugs through nanoparticles, thus making more precise and less adverse treatment possible. LncRNAs can finely and flexibly regulate gene expression processes at multiple levels, such as the regulation of transcription, translation, and processing modifications of related genes. Compared with traditional drugs, lncRNA therapy has the potential to avoid the side effects caused by drugs and achieve better efficacy. Current research on lncRNA-targeted microglia for the treatment of CNS disorders suggests that lncRNAs have achieved significant results in the treatment of neuropathic pain, epilepsy, ischemic stroke and neurodegenerative diseases and other neurological disorders. This suggests that gene precision therapy using lncRNAs is expected to replace traditional drug therapy and become a new therapeutic approach.

However, there are some limitations in this paper, the current research on lncRNAs is still relatively superficial, and the mechanism of regulation on microglia is still complicated, and the regulatory effect of H19 on microglia polarization in different environments mentioned above is heterogeneous. In order to avoid off-target effects, whether there are differences in the regulation of microglia polarization by lncRNAs under different conditions still needs a lot of research to elucidate. It is conjectured that this may be because current studies of lncRNAs are still at the stage of knocking down individual lncRNAs to study their possible functions, whereas overall behavioral changes such as depression may be the result of multiple lncRNAs under control. Also of interest are the differences in the findings of microglia *in vivo* and *in vitro*. Studies have shown that microglia are sensitive to external stimuli. Leaving the brain may lead to phenotypic and functional changes. This may be the reason why microglia in primary culture are sensitive to Aβ but no changes were observed *in vivo*. Also, *in vivo* studies may be region-specific. A related study found that IL-4 induced different M2 responses in striatal and frontal cortical microglia. This is related to the efficiency of M2a gene expression at mRNA and protein levels in different regions. Therefore, the differences in the results of the relevant studies may also be related to the site of action of lncRNA. The effect of cell therapy may also be different due to the different efficiency of gene expression at different sites. Finally, the current research on microglia regulation by lncRNAs is relatively limited by the current microglia polarization typing, and its more scientific typing and nomenclature needs to be further studied and refined.

## Author Contributions

TW designed the article and translated the article into English. XG, HT, ZC and TW wrote the manuscript. PL and WS helped research and collect the materials. XG and TW prepared figures. TW and WG critically revised the manuscript for important intellectual content. ZC, HT, WS and PL fixed some errors. All authors read and approved the final manuscript and agreed to be accountable for all aspects of this work.

## Conflict of Interest

The authors declare that the research was conducted in the absence of any commercial or financial relationships that could be construed as a potential conflict of interest.

## Publisher’s Note

All claims expressed in this article are solely those of the authors and do not necessarily represent those of their affiliated organizations, or those of the publisher, the editors and the reviewers. Any product that may be evaluated in this article, or claim that may be made by its manufacturer, is not guaranteed or endorsed by the publisher.
